# Laser‐Structured Nickel Electrodes for Enhanced Hydrogen Evolution Kinetics in Alkaline Media at Elevated Temperature

**DOI:** 10.1002/smsc.70426

**Published:** 2026-10-25

**Authors:** Mayla Selene Barrientos, Lis Geraldine Zschach, Robert Baumann, Andrés Fabián Lasagni

**Affiliations:** ^1^ Institut für Fertigungstechnik Technische Universität Dresden Dresden Germany; ^2^ Fraunhofer‐Institut für Werkstoff‐ und Strahltechnik IWS Dresden Germany

**Keywords:** catalytic activity, electrochemically active surface area, hydrogen evolution reaction, laser structuring, reaction kinetics, temperature depended

## Abstract

Tailoring the surface morphology of electrocatalysts is one potential opportunity for improving the hydrogen evolution reaction (HER) in alkaline media. Direct laser interference patterning (DLIP) emerged as a promising surface engineering technique for enhancing the HER performance of nickel electrocatalysts in alkaline media. In this study, DLIP is employed to fabricate periodic line‐like microstructures on Ni electrodes using a picosecond‐pulsed laser system (*λ* = 1064 nm, *τ* = 70 ps). By varying cumulated laser fluence up to 2.4 kJ cm^−2^, a maximum structure depth of 2.35 ± 0.04 μm and interfacial area ratio up to 87% is reached. HER is evaluated by linear sweep voltammetry (LSV) and electrochemical impedance spectroscopy (EIS) at 22 and 60 °C. The DLIP‐structured electrodes exhibit significantly enhanced HER activity compared with untreated Ni, showing overpotential reductions of up to 28% at 22 °C and 53% at 60 °C, in conjunction with an increase in electrochemically active surface area (ECSA) from 2.24 to 31.04 cm^2^ at 22 °C and from 1.95 to 74.84 cm^2^ at 60 °C, respectively. These findings highlight the favorable interplay between DLIP‐induced surface modification and thermally enhanced HER kinetics.

## Graphical abstract

Periodic microstructures fabricated by direct laser interference patterning enhance the electrochemical performance of Ni electrodes in alkaline media. Combining laser‐induced surface structuring with elevated temperature reduces HER overpotential up to 48% and increases the electrochemically active surface area from 1.95 to 74.84 cm^2^, resulting in highly efficient and stable hydrogen evolution.

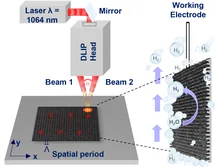

## 1 Introduction

The use of green hydrogen as an alternative energy carrier to mitigate greenhouse gas emissions and reduce dependence on fossil fuels has gained significant attention in the global energy sector, particularly in the transition toward sustainable energy systems [1, 2, 3, 4, 5, 6]. Hydrogen can be generated via water electrolysis in acidic or alkaline media using electricity which can be provided by solar and wind power, thereby reducing CO_2_ emissions [7, 8, 9, 10]. In particular, alkaline water electrolysis (AWE) has emerged as an attractive alternative for sustainable hydrogen production since the materials used to produce it are compatible with earth‐abundant catalysts, and it offers advantages in terms of cost, durability, and scalability for industrial applications [11, 12, 13].

In alkaline media, the HER begins with the electrochemical dissociation of water molecules, known as Volmer step, producing adsorbed hydrogen intermediates ($H^{*}$).

The adsorbed hydrogen intermediates subsequently form H_2_ either by electrochemical desorption in the Heyrovsky step or by chemical recombination in the Tafel step [14]. Accordingly, the overall reaction follows either the Volmer–Heyrovsky or Volmer–Tafel mechanisms, depending on the rate‐determining step. The corresponding Tafel slopes provide insight into the rate‐determining steps governing the reaction kinetics, with characteristic values of 120, 40, and 30 mV dec^−1^ associated with Volmer‐, Heyrovsky‐, and Tafel‐limited processes, respectively [15, 16, 17, 18]. However, HER kinetics in alkaline systems are limited by the additional energy barrier associated with water dissociation during the Volmer step [17]. Furthermore, water adsorption and dissociation on the catalyst surface, along with the stabilization of reaction intermediates, critically influence HER performance in alkaline media [19]. In this context, the selection and design of the electrode material are crucial for improving catalytic activity [20].

Nickel‐based materials are among the most widely investigated electrocatalysts for HER in alkaline media due to their low cost, good electrical conductivity, and chemical stability [21, 22, 23, 24, 25]. In particular, Ni‐foams and meshes serve as effective electrodes, providing a high active surface area and efficient charge transport [26]. However, their catalytic performance strongly depends on the surface morphology. In this context, Hu et al. reported an overpotentials of 217 mV at 10 mA cm^−2^ with a Tafel slope of 130 mV dec^−1^ for Ni‐foam electrodes in alkaline media [27]. Despite these advantages, the catalytic activity remains significantly lower than noble metals such as Pt [28, 29, 30].

To overcome these limitations, several surface modification techniques have been investigated to reduce overpotentials and enhance catalytic performance [29, 30, 31]. For example, Abbas et al. synthesized echinoid, or sea urchin‐like, Ni nanoparticles in ethanol using oleylamine (OAm). The samples prepared at OAm/Ni molar ratios of 6 and 4 required overpotentials of 180 and 202 mV, respectively, to reach a current density of 10 mA cm^−2^ in an alkaline electrolyte at 25 °C [32]. Similarly, Bernäcker et al. produced porous Raney Ni electrodes using a powder metallurgical process involving the deposition of Al powder onto a Ni mesh, heat treatment to form Ni–Al intermetallic phases, and subsequent alkaline leaching. The electrode prepared with 17.1 wt% Al powder relative to the Ni mesh required an overpotential of 118 mV to reach a current density of 300 mA cm^−2^ at 60 °C, representing an approximately 70% reduction relative to the 393 mV measured for the untreated Ni mesh [33].

Among these approaches, laser‐induced surface structuring has emerged as a versatile strategy for tailoring electrode morphology and surface properties [34, 35]. Laser processing techniques provide precise control over surface topography, enabling the formation of micro‐ and nanostructures. These microstructures have shown to promote electrocatalytic performance in different recent studies [36, 37, 38, 39]. For instance, Ränke et al. combined direct laser interference patterning (DLIP) with polygon‐scanner processing to fabricate hierarchical line‐like patterns on Ni foils. The surfaces consisted of parallel microscale ridges and valleys with center‐to‐center spacings of 11 and 25 µm and structure depths of up to 15 µm, together with superimposed nanoscale features. In alkaline media at 60 °C, the structured electrodes exhibited an approximately 22% lower HER overpotential than untreated Ni electrodes. In other work, Rauscher et al. employed an ultrashort‐pulsed laser with a pulse duration of 60 fs and a wavelength of 800 nm, also to modify the surface of Ni‐electrodes, producing laser‐induced periodic surface structures (LIPSS). They reported an overpotential reduction of approximately 45% for HER in 29.9 wt% KOH at ~60 °C relative to smooth Ni surfaces [37].

The improvements found in these studies are mainly attributed to the ability of laser processing to modify the surface roughness. In particular, the increased surface roughness and the formation of hierarchical surface micro/nanostructures promote electrolyte penetration and facilitate hydrogen bubble detachment, further enhancing mass‐transport processes under HER conditions [36, 37, 38, 39, 40]. For instance, Piscitelli et al. investigated the HER performance and hydrogen bubble dynamics on DLIP‐structured nickel electrodes in alkaline media. Using nanosecond laser pulses at 355 nm, the authors reported a remarkable decrease of 55 mV in the overpotential required to achieve a current density of 100 mA cm^−2^, which was attributed to improved hydrogen bubble dynamics [40].

In our previous work, DLIP‐generated line‐like structures on Ni electrodes were shown to modify the surface topography, wettability, surface chemistry, and electrochemically active surface area, with the electrochemical response evaluated at 22 and 60 °C [39]. However, that study focused primarily on the surface properties and capacitive behavior of the electrodes and did not directly quantify the HER polarization behavior, apparent reaction kinetics, interfacial charge transfer, or electrochemical stability. The present work therefore builds on the previous investigation using newly fabricated Ni electrodes produced under the same selected DLIP processing conditions, focusing on the electrocatalytic behavior under relevant industrial conditions. Linear sweep voltammetry, Tafel analysis, electrochemical impedance spectroscopy, chronoamperometry, and chronopotentiometry are employed at 22 and 60 °C to determine whether the previously observed increases in surface development and electrochemical accessibility translate into lower HER overpotentials, reduced charge‐transfer resistance, and stable electrochemical behavior during the investigated tests.

## 2 Results and Discussion

### 2.1 Laser Surface Structuring

Based on the processing window established in our previous study [39], three process conditions were selected to produce Ni electrodes with systematically varied surface topographies. Fresh electrode specimens were fabricated for the present investigation and independently characterized by SEM and WLI before electrochemical testing. The resulting topographical parameters therefore correspond to the electrodes used for the HER kinetic, impedance, and stability measurements reported here.

Laser structuring of nickel surface electrodes was performed using the DLIP technique. By interfering two laser beams, periodic line‐like structures, with a spatial period (Λ_DLIP_) of 5.4 μm were produced at different cumulated laser fluences (Φ_cum_) (see Experimental Section). The Φ_cum_ denotes the total laser energy delivered per unit area and was calculated from the laser pulse energy (*E*_p_), the applied number of pulses per unit of area (*N*_pulses_), the laser spot area (*A*_spot_), and the repetition rate of the laser source (*f*), as described by Equations (S1)–(S4). The laser‐processing parameters are also summarized in Table S1.

For qualitative analysis, SEM images of the reference Ni electrode and the fabricated DLIP line‐like structures were taken and are presented in Figure 1. The untreated nickel surface shown in Figure 1a exhibits an irregular topography, characteristic of the used foil. At the lowest cumulative fluence (Φ_cum_ = 48.8 J cm^−2^), the surface exhibits a weakly developed periodic line‐like pattern with a spatial period of 5.4 µm, corresponding to the imposed DLIP intensity distribution (Figure 1b). The limited topographical modulation at this fluence is in agreement with the relative low fluences applied [35, 39, 41, 42]. In addition, finer periodic features are additionally observed within the DLIP‐patterned regions and are consistent with the formation of laser‐induced periodic surface structures (LIPSS) [43].

**FIGURE 1:**
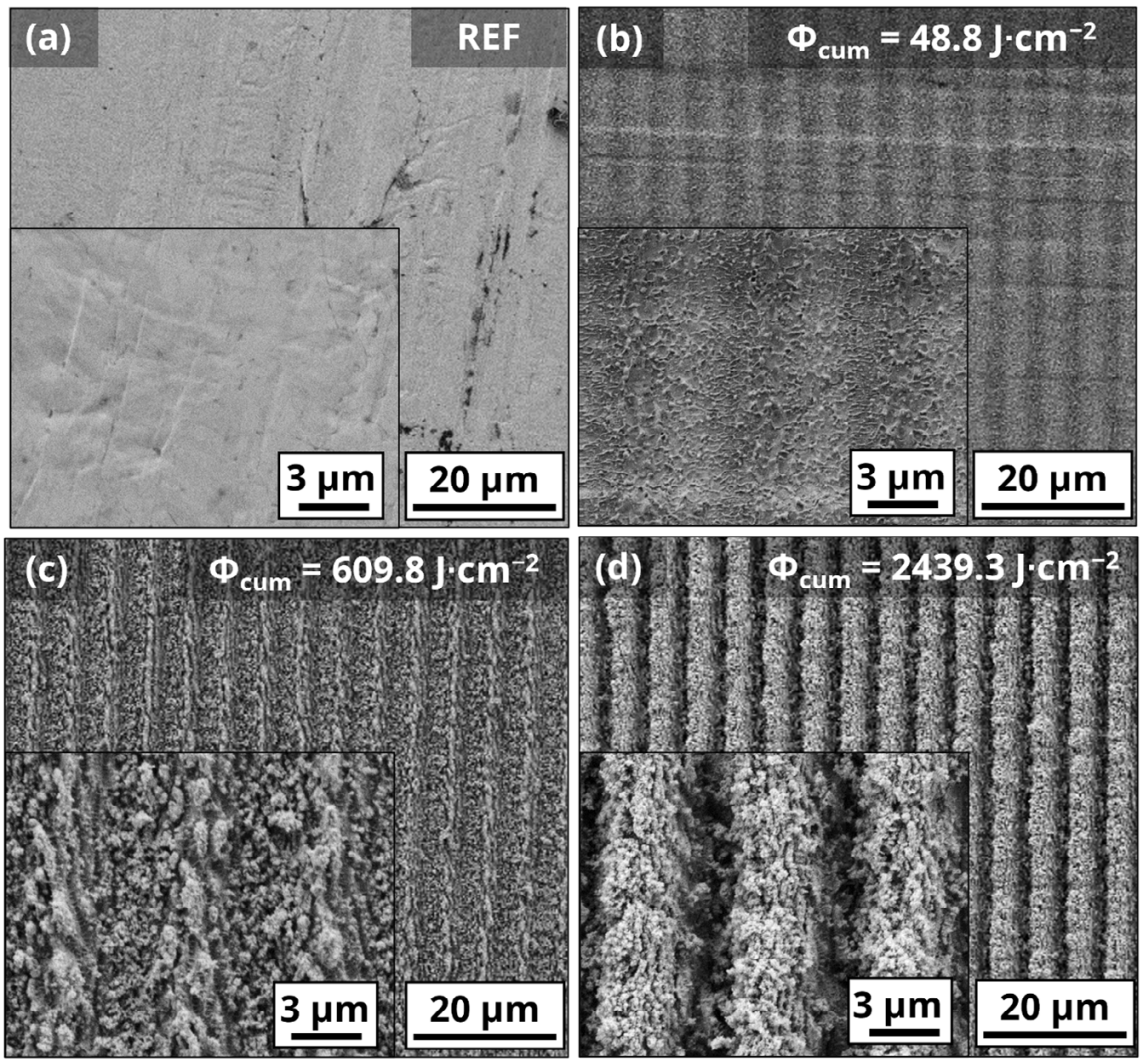
SEM images of (a) unstructured reference Ni foil and line‐like DLIP periodic Ni structured with cumulated fluence of (b) 48.8 J cm^−2^, (c) 609.8 J cm^−2^, and (d) 2439.3 J cm^−2^. The resulting periodic structures reveal a spatial period (Λ_DLIP_) of 5.4 μm.

Subsequently, when the cumulated fluence is increased to 609.8 J cm^−2^, the surface exhibits a more defined line‐like periodic structure (see Figure 1c). A further increase to 2439.3 J cm^−2^, achieved by applying a larger number of laser pulses per unit area, produces pronounced and well‐defined ridges and valleys, as shown in Figure 1d.

For further evaluation of the surface topography, the laser‐structured Ni electrodes were quantitatively characterized by white light interferometry (WLI). Representative surface height maps obtained from an area of 150 × 150 μm^2^ for each electrode are presented in Figure 2. The corresponding roughness parameters extracted prior and after electrochemical characterization are summarized in Table 1, including the structure depth (*S*_d_) of the DLIP structures, the arithmetical mean height (*S*_a_), the maximum height (*S*_z_), and the developed interfacial area ratio (*S*_dr_).

**FIGURE 2:**
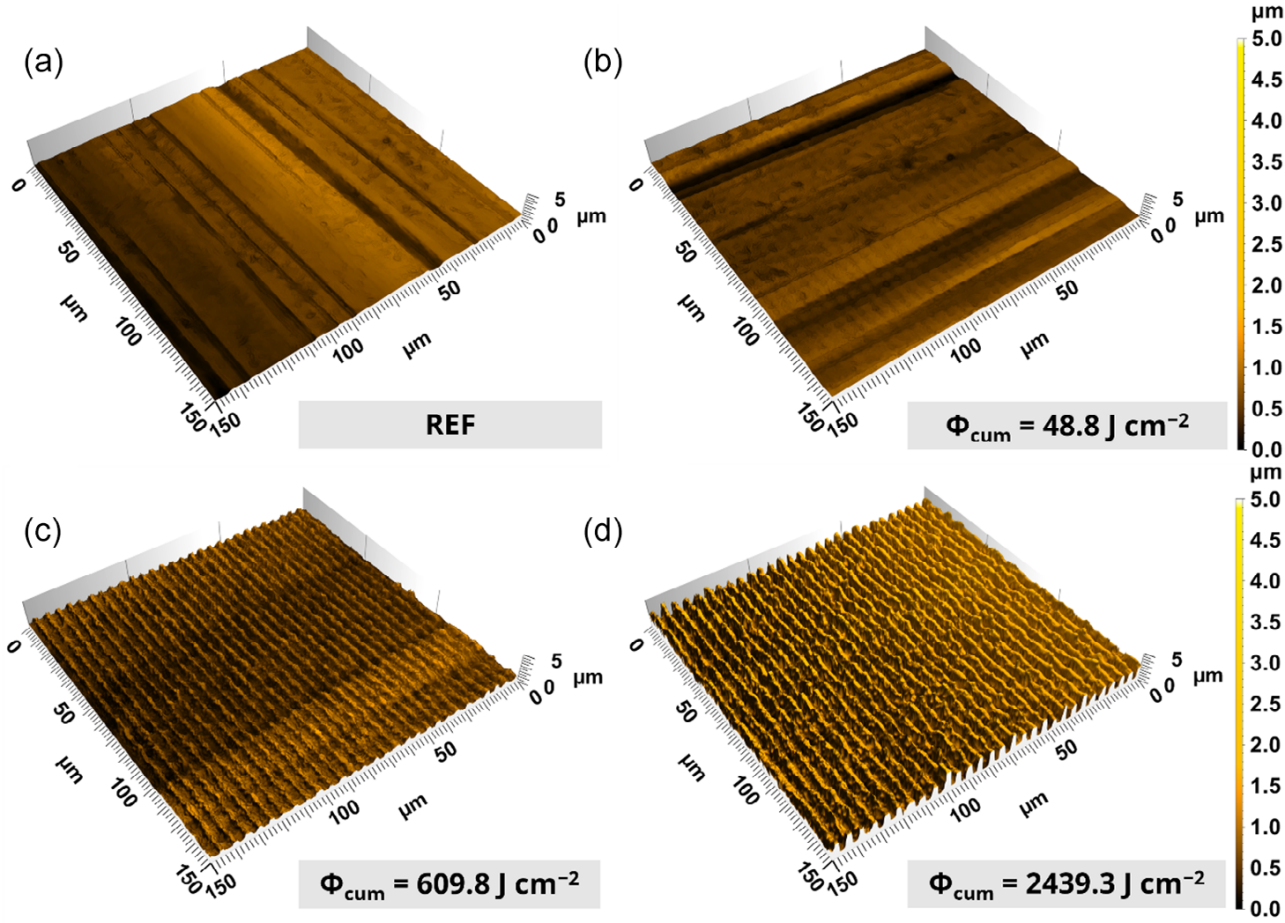
WLI surface height maps of (a) the untreated reference Ni surface and Ni foils patterned with line‐like DLIP structures at cumulative fluences of (b) 48.8 J cm^−2^, (c) 609.8 J cm^−2^, and (d) 2439.3 J cm^−2^. All DLIP‐patterned surfaces exhibit a spatial period (Λ_DLIP_) of 5.4 μm.

**TABLE 1:** Surface topography parameters derived from the WLI surface analysis for reference Ni foil and line‐like DLIP structured Ni prior and after electrochemical characterization.

	Cum. fluence Φ_cum_, J cm^−2^	Structure depth *S*_d_, μm	Arithmetical mean height *S*_a_, μm	Maximum height *S*_z_, μm	Interfacial area ratio *S*_dr_, %
Prior electrochemical analysis	0 (Ref)	0.25 ± 0.00	0.23 ± 0.03	2.13 ± 0.32	0.80 ± 0.06
	48.8	0.24 ± 0.01	0.14 ± 0.03	1.33 ± 0.07	0.79 ± 0.10
	609.8	0.68 ± 0.01	0.25 ± 0.02	1.95 ± 0.18	11.37 ± 0.29
	2439.3	2.35 ± 0.04	0.81 ± 0.01	4.26 ± 0.14	87.29 ± 0.21
After electrochemical analysis	0 (Ref)	0.29 ± 0.05	0.17 ± 0.03	1.53 ± 0.16	1.17 ± 0.26
	48.8	0.26 ± 0.03	0.26 ± 0.07	1.82 ± 0.39	1.14 ± 0.16
	609.8	0.59 ± 0.09	0.26 ± 0.01	2.10 ± 0.24	11.13 ± 1.23
	2439.3	2.51 ± 0.05	0.88 ± 0.01	5.44 ± 0.43	107.33 ± 6.11

The untreated reference nickel foil, shown in Figure 2a, exhibits a strongly directional surface topography, characterized by parallel grooves, originating from the foil manufacturing process of the commercial used material (as discussed before). The measured roughness values were *S*_a_ = 0.23 ± 0.03 μm and *S*_z_ = 2.13 ± 0.32 μm (Table 1).

At the lowest cumulative fluence of 48.8 J cm^−2^, the DLIP structure depth was 0.24 ± 0.01 μm, while *S*_a_ and *S*_z_ slightly decreased to 0.14 ± 0.03 and 1.33 ± 0.07 μm, respectively (Figure 2b). This reduction suggests that the initial laser treatment partially attenuated the original surface irregularities while introducing a shallow periodic modulation due to the DLIP treatment. The corresponding *S*_dr_ value of 0.79% ± 0.01% indicates only a minor increase in the developed surface area.

Increasing the cumulative fluence to 609.8 J cm^−2^ increased the structure depth of the DLIP features *S*_d_ to 0.68 ± 0.007 μm and *S*_dr_ to 11.37% ± 0.29% (Figure 2c). The values of *S*_a_ and *S*_z_ also increased to 0.25 ± 0.02 and 1.95 ± 0.18 μm, respectively. At the highest cumulative fluence of 2439.3 J cm^−2^, the topographical parameters reached their maximum values, with *S*_d_ = 2.35 ± 0.04 μm, *S*_a_ = 0.81 ± 0.01 μm, *S*_z_ = 4.26 ± 0.14 μm, and *S*_dr_ = 87.29% ± 0.21% (Figure 2d). The increase in structure depth and developed interfacial area with cumulative fluence is consistent with previous studies [39].

Based on the employed laser source and DLIP optic (Elipsys Surfunction) and given process parameters of 0.7 mm s^−1^ and a 358‐µm spot size, the deepest structure depths could be manufactured at a rate of 15.05 mm^2^ min^−1^. Previous works already showed the capability of reaching over 1 m^2^ min^−1^ by using a different experimental setup [44]. Nevertheless, maximizing the structuring throughput was out of the scope of this described experiment, which focus more on the interplay of the DLIP texture and chemical reaction kinetics for HER.

These controlled topographical variations therefore provide the basis for assessing their influence on HER kinetics and interfacial charge transfer at elevated temperatures.

### 2.2 HER Activity and Interfacial Charge‐Transfer Analysis

To evaluate the electrocatalytic activity of the DLIP‐structured Ni electrodes toward HER in alkaline media, polarization curves recorded by linear sweep voltammetry (LSV) were performed in 1 M NaOH at two temperatures (22 and 60 °C) following the protocol described in the Experimental Section. LSV was conducted in the range from 0.0 to −1.3 V (vs. Hg/HgO), and the corresponding Tafel analysis was employed to assess the reaction kinetics. The resulting polarization and Tafel plots are presented in Figure 3.

**FIGURE 3:**
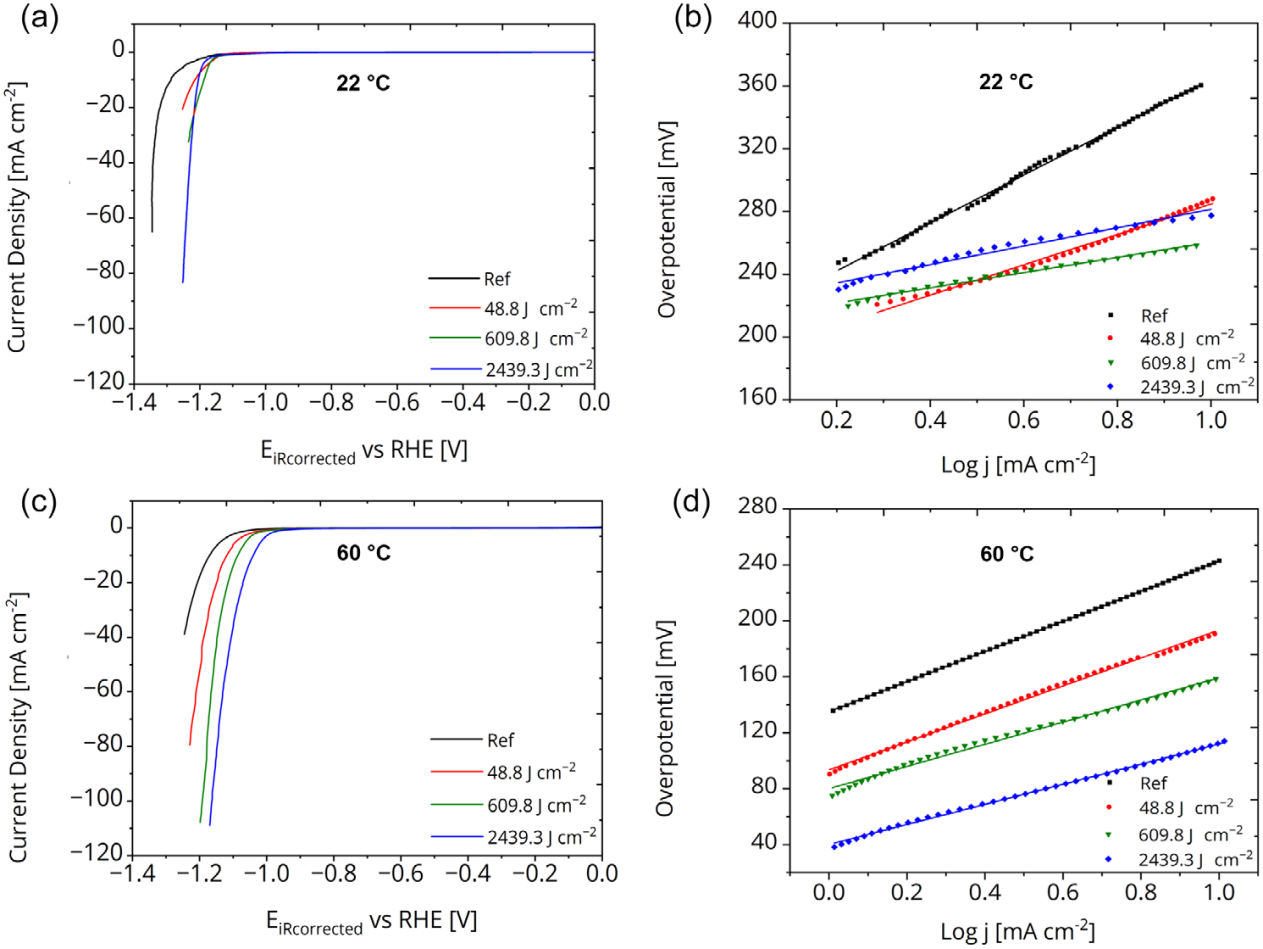
Linear sweep voltammetry (LSV) curves and the corresponding Tafel slopes of the unstructured reference and the DLIP‐structured Ni foils with cumulated fluences of 48.8, 609.8, and 2439.3 J cm^−2^ in NaOH 1 M at (a, b) 22 °C and (c, d) 60 °C (iR‐corrected using the solution resistance (*R*_s_) extracted from EIS measurements).

For a consistent comparison of catalytic performance, all potentials were converted from the Hg/HgO reference electrode to the reversible hydrogen electrode (RHE) scale as follows:

(1) $$E_{\text{RHE}}=E_{\mathrm{Hg}/\mathrm{HgO}}^{0}+\frac{2.303\cdot RT}{F}\cdot \mathrm{pH}+E_{\mathrm{Hg}/\mathrm{HgO}},$$

where *R* is the universal gas constant (8.314 J mol^−1^ K^−1^), *T* is the temperature, *F* is the Faraday constant (96485 C mol^−1^), and *E* ^0^_Hg/HgO_ is the standard potential of the Hg/HgO electrode (0.098 V at 25 °C) [45, 46, 47, 48, 49, 50].

The HER activity of the unstructured and DLIP‐structured Ni electrodes at 22 °C is presented in Figure 3a. The LSV curves exhibit a clear shift toward lower overpotentials after laser structuring, indicating enhanced catalytic activity compared with the untreated reference surface. This tendency is further analyzed through the corresponding Tafel plots shown in Figure 3b. The overpotential (*η*) was determined as the difference between the applied potential and the thermodynamic equilibrium potential for HER [51], according to:

(2) $$\text{η}=E_{\text{RHE}}-{\text{E}}_{\text{eq}}\text{,}$$

where *E*_RHE_ is the applied potential and *E*_eq_ corresponds to the equilibrium potential for HER [32, 52]. In addition, the Tafel slope was determined from the electrochemical data using the Tafel equation as follows:

(3) $$\eta =a+b\cdot \log_{i},$$

where *a* is the intercept and *b* is the Tafel slope [53].

The overpotential and the Tafel slope determined at a current density of 10 mA cm^−2^ are summarized in Table 2, together with the regression statistics of the Tafel fit, represented by the coefficient of determination *R* ^2^.

**TABLE 2:** Electrochemical HER parameters of the unstructured and DLIP‐structured Ni electrodes measured in 1 M NaOH at 22 and 60 °C (iR‐corrected using the solution resistance (*R*_s_) extracted from EIS measurements).

22 °C				60 °C			
Φ_cum_, J cm^−2^	Overpotential at 10 mA, cm^−2^, mV	−b, mV dec^−1^	*R* ^2^, −	Overpotential at 10 mA cm^−2^, mV		−b, mV dec^−1^	*R* ^2^, −
0 (Ref)	360	152 ± 1.0	0.99	243		108 ± 0.1	0.99
48.8	288	97 ± 1.0	0.98	192		99 ± 0.7	0.99
609.8	260	48 ± 1.0	0.99	160		79 ± 1.0	0.99
2439.3	277	58 ± 1.0	0.98	114		71 ± 0.4	0.99

The calculated values for the curves recorded at 22 °C show that the unstructured Ni electrode requires an overpotential (*η*_10_) of 360 mV to reach 10 mA cm^−2^, whereas the DLIP‐structured electrodes exhibit markedly improved performance, with *η*_10_ values of 288 mV (Φ_cum_ = 48.8 J cm^−2^), 260 mV (Φ_cum_ = 609.8 J cm^−2^), and 277 mV (Φ_cum_ = 2439.3 J cm^−2^). These results correspond to a reduction in the overpotential up to ~100 mV relative to the reference electrode.

In addition, the reference electrode exhibits a high Tafel slope of 152 ± 1 mV dec^−1^, characteristic of slow HER kinetics in alkaline media and consistent with a Volmer mechanism, where water dissociation plays a major role in limiting the reaction [14]. In contrast, the DLIP‐treated electrodes significantly reduced the Tafel slope to 97 ± 1 mV dec^−1^ (Φ_cum_ = 48.8 J cm^−2^), 48 ± 1 mV dec^−1^ (Φ_cum_ = 609.8 J cm^−2^), and 58 ± 1 mV dec^−1^ (Φ_cum_ = 2439.3 J cm^−2^), indicating a more favorable HER reaction kinetics and a possible shift toward a Volmer–Heyrovsky pathway [14, 15]. This behavior is widely reported for advanced nanostructured Ni catalysts [28]. Notably, the electrode processed at the Φ_cum_ 609.8 J cm^−2^ exhibits the lowest Tafel slope, despite showing comparable *η*_10_ values to the 2439.3 J cm^−2^ sample, indicating improved kinetic behavior within the selected Tafel region, although the overpotential at 10 mA cm^−2^ remains essentially similar for both conditions.

Relating the electrochemical response to the measured topographical parameters shows that the improvement in HER activity cannot be explained solely by the microscale surface area determined by WLI. The untreated reference and the electrode processed at 48.8 J cm^−2^ exhibit similar values of *S*_d_ and *S*_dr_; nevertheless, the DLIP treatment *η*_10_ from 360 to 288 mV and the Tafel slope from 152 to 97 mV dec^−1^. This improvement may therefore also involve the finer LIPSS observed by SEM, which is not fully resolved by WLI. Increasing the cumulative fluence to 609.8 J cm^−2^ raises *S*_d_ to 0.68 μm and *S*_dr_ to 11.37%, accompanied by a further decrease in *η*_10_ to 260 mV and in the Tafel slope to 48 mV dec^−1^. However, the substantially larger *S*_dr_ of 87.29% obtained at 2439.3 J cm^−2^ does not produce an additional reduction in *η*_10_ at 22 °C, indicating that the influence of surface development on the geometrically normalized HER response approaches a plateau under these conditions.

For investigating the influence of temperature, the electrocatalytic performance was further evaluated at 60 °C, as presented in Figure 3c,d. Compared to the measurements at 22 °C, the shift to lower overpotentials in the polarization curves is more pronounced, indicating accelerated HER kinetics at elevated temperatures. This effect is more pronounced for the DLIP‐structured electrodes than for the reference sample, suggesting that laser‐induced surface structuring enhances the electrocatalytic HER performance also at higher temperatures, in agreement with previous studies on laser‐structured electrodes [35, 36, 37].

As summarized in Table 2, *η*_10_ decreases from 243 mV for the unstructured reference electrode to 114 mV for the electrode processed with the highest Φ_cum_ of 2439.3 J cm^−2^. This corresponds to an overpotential reduction of 129 mV, or approximately 53%, relative to the reference, identifying this electrode as the most active under the evaluated conditions.

The influence of temperature on the HER is further supported by Tafel analysis, presented in Figure 3d. The slope of the reference sample decreases from 152 at 22 °C to 108 mV dec^−1^ at 60 °C, indicating that the kinetic limitations associated with water dissociation are partially reduced at elevated temperature [14]. However, the slope suggests predominantly Volmer‐path kinetics govern the electrochemical behavior of the unstructured reference also at high temperature [29]. In contrast, the DLIP‐structured electrodes exhibit consistently lower Tafel slopes with increasing cumulated fluence, reaching values of 99 mV dec^−1^ (Φ_cum_ = 48 J cm^−2^), 79 mV dec^−1^ (Φ_cum_ = 609.8 J cm^−2^), and 71 mV dec^−1^ (Φ_cum_ = 2439 J cm^−2^). This trend indicates that at high temperatures, the HER reaction kinetics also shift toward a Volmer–Heyrovsky path, confirming that DLIP‐structuring, combined with the elevated temperature, promotes alkaline HER by facilitating water dissociation and interfacial charge‐transfer process [16, 54, 55, 56, 57]. These results are in good agreement with several studies demonstrating that nanostructured nickel surfaces enhance alkaline HER activity by modifying surface properties, thereby reducing the overpotential and the Tafel slope [28, 36, 57, 58]. Collectively, the Ni‐treated sample using the highest cumulated fluence (Φ_cum_ = 2439 J cm^−2^) presented the most accelerated HER kinetic, in addition to the highest ECSA (see Section 2.3). Moreover, the observed activity enhancement is consistent with our previous findings for Ni surfaces processed under equivalent DLIP conditions, which exhibited improved wettability and higher surface fractions of Ni oxide and hydroxide species at high cumulated fluences [39].

Finally, it is important to note that the overpotential required to drive the HER is governed not only by intrinsic catalytic activity, but also by resistive and interfacial charge‐transfer contributions arising from the electrode/electrolyte interface [19, 29]. Therefore, to further elucidate these contributions, EIS measurements were performed. The corresponding Nyquist and Bode plots for the EIS measurements performed at 200 mV at both temperatures are presented in Figure 4 together with the respective equivalent electrical circuit (EEC) used to model their electrochemical behavior.

**FIGURE 4:**
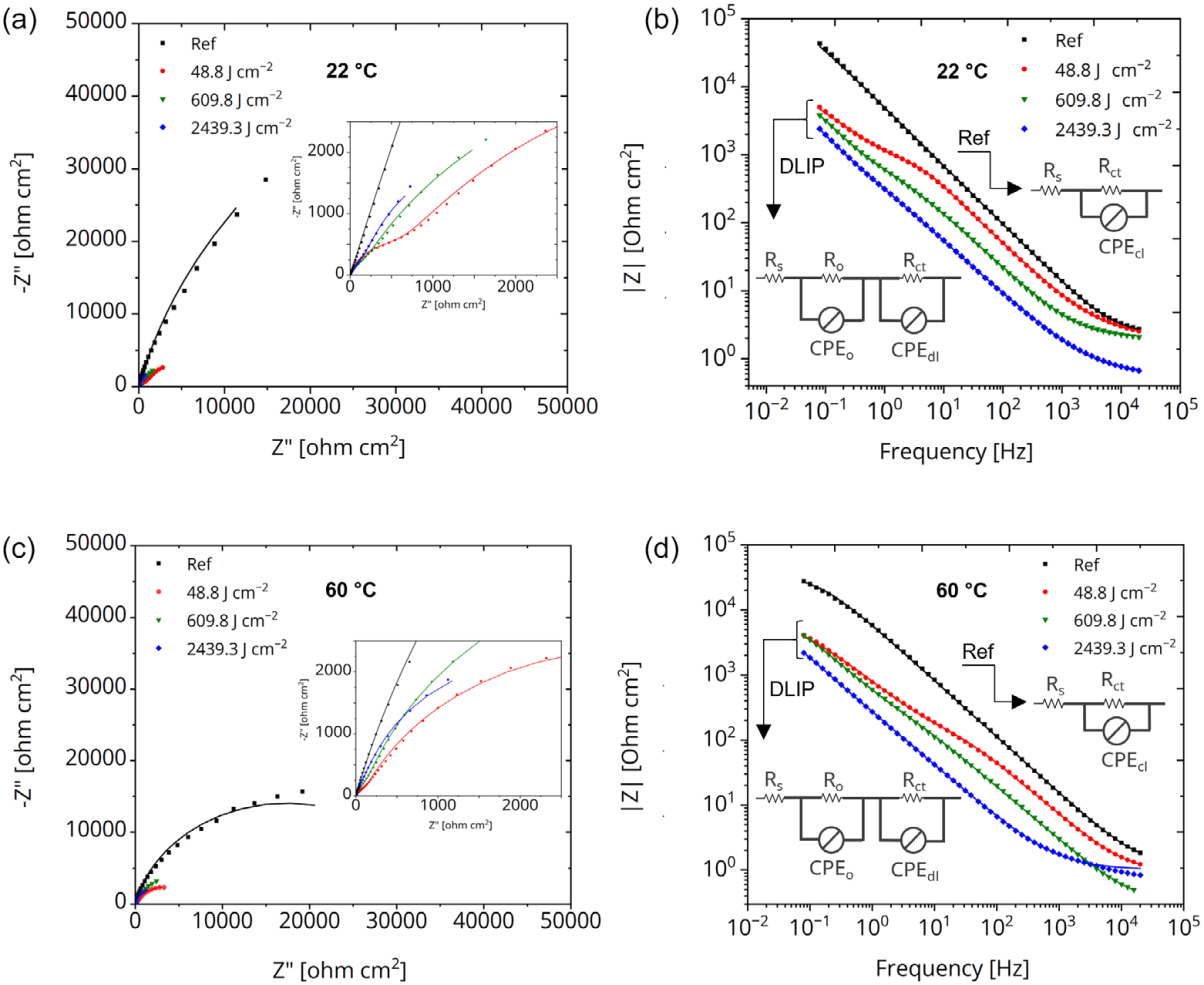
Nyquist and Bode plots obtained from EIS measurements at a potential of 200 mV for the unstructured reference and the DLIP‐structured Ni electrodes in 1 M NaOH, at (a, b) 22 °C and (c, d) 60 °C. The corresponding equivalent electrical circuits used for fitting the impedance spectra are also included.

The EIS response of the reference Ni electrode was fitted using a Randles equivalent circuit comprising the solution resistance (*R*_s_) in series with a parallel combination of the charge‐transfer resistance (*R*_ct_) and a constant phase element representing the non‐ideal double‐layer capacitance (CPE_cl_). In contrast, the DLIP‐structured electrodes were analyzed using an equivalent circuit with two‐time constants, defined by *R*_o_ and CPE_o_ as well as *R*_ct_ and CPE_cl_, which is commonly used to describe porous electrode surfaces [37, 59, 60].

The resistive contributions to the electrochemical reaction arise from the oxide layer resistance R_o_ and the charge‐transfer resistance *R*_ct_. The high‐frequency response is attributed to interfacial capacitive processes at the electrode/electrolyte interface, represented by a constant phase element (CPE_o_). The low‐frequency processes are associated with the HER charge‐transfer resistance between the electrode and the electrolyte (*R*_ct_ with CPE_cl_), which may be influenced by the presence of surface Ni oxide/hydroxide species, commonly observed on nickel electrodes under alkaline conditions [60, 61].

At 22 °C, the Nyquist plots, shown in Figure 4a, exhibit depressed semicircles whose diameters decrease with increasing laser fluence, indicating enhanced electrocatalytic activity. Similarly, the corresponding Bode plots presented in Figure 4b show a systematic decrease in impedance modulus (|*Z*|) across the entire frequency range.

At 0.01 Hz, |*Z*| decreases from 35 970 Ω cm^2^ for the reference to 1955 Ω cm^2^ for the laser‐structured sample at Φ_cum_ = 2439.3 J cm^−2^. The lower impedance modulus accompanied by the smaller semicircles observed in the Nyquist plot indicates that the treated samples exhibit reduced interfacial resistance to HER. This trend is consistent with the fitted values of the EEC reported in Table S3.

At 60 °C, a similar trend is observed, with a further reduction in the diameter of the Nyquist semicircles (Figure 4c), indicating lower overall resistances. The Bode plot (Figure 4d) also reveals lower impedance modulus, where |*Z*| at 0.1 Hz decreases from 24 820 Ω cm^2^ for the reference to 1831 Ω cm^2^ in the case of the laser‐structured sample at the highest laser fluence (Φ_cum_ = 2439.3 J cm^−2^). The fitted values for these samples are presented in Figure S3. The reduced resistive behavior is associated with more efficient charge transfer at the electrode/electrolyte interface and supports the enhanced HER kinetics inferred from the Tafel analysis.

To further evaluate the HER behavior with varying the applied potential, EIS measurements were performed at 100 and 300 mV, also at both temperatures. The corresponding Nyquist and Bode plots are presented in the Supporting Information (Figures S1 and S2), along with the fitted values (Tables S2*–*S4). The variation in *R*_ct_ values across the tested potentials (100, 200, and 300 mV) at both temperatures is presented in Figure 5.

**FIGURE 5:**
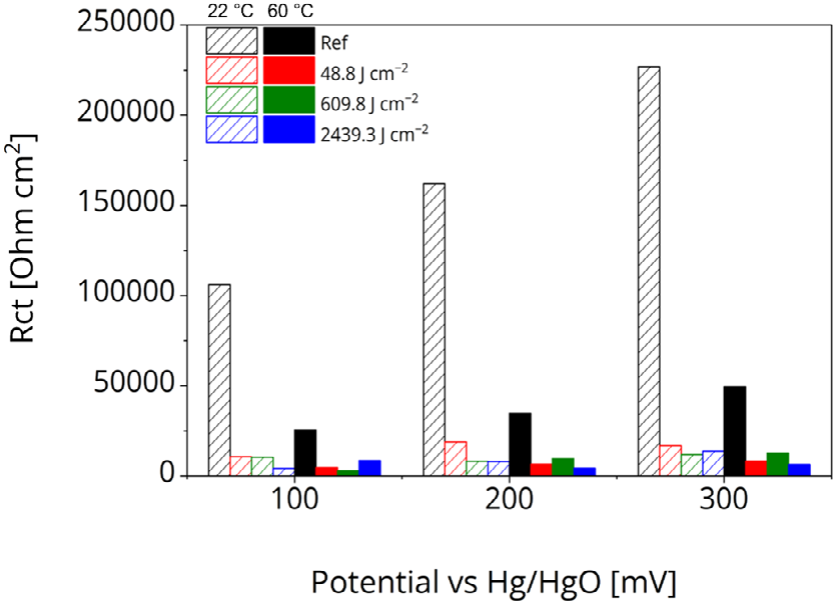
Evolution of the charge transfer resistance (*R*_ct_) as a function of applied potential for the unstructured reference and the DLIP‐structured Ni electrodes measured in 1 M NaOH at 22 and 60 °C.

A clear trend is observed across all investigated potentials in Figure 5, characterized by a significant decrease in *R*_ct_ for the textured DLIP electrodes. At both 22 and 60 °C, it is observed that the reference electrode consistently exhibits the highest charge‐transfer resistance, while the DLIP‐structured electrodes show substantially reduced *R*_ct_ values. Under a low applied potential of 100 mV at 22 °C, *R*_ct_ exhibits a strong dependence on surface structuring. The reference electrode shows a high *R*_ct_ value of 106 000 Ω cm^2^. In contrast, the DLIP‐structured electrodes display significantly lower values, decreasing to 10 800 Ω cm^2^ (Φ_cum_ = 48.8 J cm^−2^) and 10 500 Ω cm^2^ (Φ_cum_ = 609.8 J cm^−2^), and reaching a minimum of 4260 Ω cm^2^ (Φ_cum_ = 2439.3 J cm^−2^); see Table S2. This reduction corresponds to an improvement of more than one order of magnitude in charge‐transfer kinetics, highlighting the favorable effect of laser‐induced surface structuring on the electrode/electrolyte interfacial properties. At 200 mV, the DLIP‐structured electrodes continue to exhibit significantly lower *R*_ct_ values than the reference electrode, reaching 18 900 Ω cm^2^ for Φ_cum_ = 48.8 J cm^−2^, while the samples processed at higher cumulated fluences display similar and markedly lower values of approximately ~8000 Ω cm^2^ (Φ_cum_ = 609.8 J cm^−2^ and 2439.3 J cm^−2^); see Table S3. At 300 mV, a further increase in *R*_ct_ is observed for all electrodes. The reference electrode reaches 227 000 Ω cm^2^, while the DLIP‐structured electrodes exhibit values of 16 800 Ω cm^2^ (Φ_cum_ = 48.8 J cm^−2^), 11 800 Ω cm^2^ (Φ_cum_ = 609.8 J cm^−2^), and 13 900 Ω cm^2^ (Φ_cum_ = 2439.3 J cm^−2^).

The influence of temperature is particularly evident on the reference electrode. A pronounced decrease in *R*_ct_ with increasing temperature is observed for each applied potential for the reference, while for the DLIP‐structured electrodes, it is less pronounced. At 60 °C, the reference electrode shows a value of 25 540 Ω cm^2^, while the DLIP‐structured electrodes exhibit markedly lower values of 4730 Ω cm^2^ (Φ_cum_ = 48.8 J cm^−2^), 2954 Ω cm^2^ (Φ_cum_ = 609.8 J cm^−2^), and 8614 Ω cm^2^ (Φ_cum_ = 2439.3 J cm^−2^); see Table S3. Increasing the applied potential to 200 mV revealed an increase in *R*_ct_ values. Notably, the electrode treated at Φ_cum_ = 609.8 J cm^−2^ deviates from the expected trend, showing a higher *R*_ct_ value of 9649 Ω cm^2^. Lastly, at 300 mV, the reference electrode reaches an *R*_ct_ of 49 400 Ω cm^2^, while the DLIP‐structured electrodes exhibit values of 8280 Ω cm^2^ (Φ_cum_ = 48.8 J cm^−2^), 12 900 Ω cm^2^ (Φ_cum_ = 609.8 J cm^−2^), and 6503 Ω cm^2^ (Φ_cum_ = 2439.3 J cm^−2^). The decrease in *R*_ct_ values indicates enhanced interfacial charge‐transfer kinetics after DLIP structuring and is consistent with the increased structure depth and developed interfacial area of the treated electrodes. Laser‐induced changes in wettability and Ni oxide/hydroxide surface species, previously observed under equivalent processing conditions, may also contribute to this behavior.

In summary, the EIS results indicate that DLIP‐induced surface structuring significantly affects the electrochemical response of the electrodes during the HER. As the applied potential increases from 100 to 300 mV, the changes in *R*_ct_ suggest that the HER response is influenced by additional interfacial factors [14, 62, 63, 64, 65, 66]. Temperature further enhances HER kinetics across all conditions, as evidenced by the systematic reduction in *R*_ct_ at 60 °C [67].

### 2.3 ECSA Determination

Following the contribution of the charge‐transfer resistance, parameters extracted from the EIS fitting, as the solution resistance (*R*_s_) and the capacitance (CPE) elements, were further evaluated. These parameters were used to determine both the double layer capacitance (*C*_dl_) and, consequently, the electrochemically active surface area (ECSA). *C*_dl_ values were calculated according to:

(4) $${\text{C}}_{\text{dl}}={\left[\text{CPE}{\left(R_{s}^{-1}+R_{p}^{-1}\right)}^{\left(\alpha -1\right)}\right]}^{1/\alpha },$$

where *α* describes the deviation from ideal capacitive behavior (*α* = 1 corresponds to a pure capacitor).

The ECSA values were subsequently determined as follows:

(5) $$\mathrm{ECSA}=\frac{C_{\mathrm{dl}}}{C_{s}},$$

where *C*_s_ represents the specific capacitance of a flat surface of the same material. The apparent ECSA was estimated using a literature‐reported specific capacitance of *C*_s_ = 40 μF cm^−2^ for Ni electrodes in alkaline media [68].

The results for all used electrodes at different potentials (100, 200, and 300 mV) and temperatures (22 and 60 °C) are summarized in Table 3.

**TABLE 3:** Double layer capacitance (*C*_dl_) and electrochemically active surface area (ECSA) from EIS measurements at different potentials (100, 200, and 300 mV) and temperatures (22 and 60 °C) for the reference untreated Ni and the DLIP‐structured electrodes with Φ_cum_ from 48.8 to 2439.3 J cm^−2^.

22 °C							60 °C					
	100 mV		200 mV		300 mV		100 mV		200 mV		300 mV	
**Φ**_**cum**_ **, J cm** ^**−2**^	*C*_dl_, µF	ECSA, cm^2^	*C*_dl_, µF	ECSA, cm^2^	*C*_dl_, µF	ECSA, cm^2^	*C*_dl_, µF	ECSA, cm^2^	*C*_dl_, µF	ECSA, cm^2^	*C*_dl_, µF	ECSA, cm^2^
0 (Ref)	89.7	2.24	76.17	1.90	76.67	1.92	78.1	1.95	46.29	1.16	51.55	1.29
48.8	123.2	3.08	80.58	2.01	84.86	2.12	177.4	4.43	161.79	4.04	131.42	3.28
609.8	537.7	13.44	460.00	11.50	307.92	7.69	492.0	12.30	505.34	12.63	258.00	6.45
2439.3	1241.7	31.04	1113.52	27.84	797.12	19.93	2993.7	74.84	1949.53	48.74	1782.38	44.55

The ECSA values presented in Table 3 exhibit a clear dependence on cumulated laser fluence, consistent with the corresponding increase in structure depth and developed interfacial area of the DLIP‐structured electrodes. The *C*_dl_ values, and consequently the ECSA for the reference electrode, exceed the geometric area (1 cm^2^), with values around 2 cm^2^ across the investigated conditions. In contrast, the DLIP‐structured electrodes show a pronounced increase in the ECSA under the investigated temperatures and applied potentials. At 22 °C and 100 mV, the ECSA increases to 3.08, 13.44, and 31.04 cm^2^ for Φ_cum_ = 48.8 J cm^−2^, Φ_cum_ = 609.8 J cm^−2^, and Φ_cum_ = 2439.3 J cm^−2^, respectively. Thus, the increase in electrochemically accessible area became particularly pronounced for the electrodes with the most strongly developed DLIP topographies [39, 69].

A similar trend is observed at 60 °C for the same potential, where the ECSA further increases, reaching a maximum value of 74.84 cm^2^ for the highest cumulated fluence of Φ_cum_ = 2439.3 J cm^−2^. This behavior can be attributed to the combined effect of the DLIP‐induced surface morphology with the elevated temperature, which together improve electrolyte accessibility and facilitate interfacial charge‐transfer processes [39].

At higher potentials (200 and 300 mV), a similar behavior at both temperatures is observed, and the ECSA increases with increasing the cumulated fluence. However, an important feature is the decrease in ECSA with increasing potential (from 100 to 300 mV) for all investigated electrodes. For instance, at 22 °C and Φ_cum_ = 2439.3 J cm^−2^, the ECSA decreases from 31.04 cm^2^ (100 mV) to 27.84 cm^2^ (200 mV) and further to 19.93 cm^2^ (300 mV). A similar trend is observed at 60 °C, where the ECSA decreases from 74.84 to 48.74 cm^2^ and 44.55 cm^2^ over the same potential range. This apparent reduction in ECSA is most likely associated with changes in the effective interfacial capacitance under HER operating conditions, including potential‐dependent double‐layer structure, surface coverage by reaction intermediates, and gas bubble effects [14, 59, 70]. A similar behavior has been reported by Dong et al. for porous Ni_3_Al electrodes in HER, where an increase in *C*_dl_ derived from EIS measurements was associated with a larger ECSA, attributed to the high surface porosity [71].

The ECSA results are consistent with the impedance analysis results, especially at 100 mV. The increase in ECSA with cumulated laser fluence (and thus with the interfacial area ratio; see Table 1) corresponds to a decrease in *R*_ct_, indicating that HER activity is governed by the availability of active sites and improved interfacial charge transfer. However, this behavior becomes less pronounced at higher applied potentials, where the interfacial processes described before contribute in a higher proportion to the HER response.

The improved HER performance of the DLIP‐structured electrodes is consistent with the combined effects of laser‐induced surface topography and surface chemical modification. The DLIP process increases the structure depth and developed interfacial area, thereby providing a larger electrochemically accessible surface, as reflected by the apparent ECSA values determined in this study. Moreover, as demonstrated by XPS analysis in our previous study using identically processed Ni surfaces under equivalent DLIP conditions, laser treatment promotes the formation of Ni oxide and hydroxide species [39]. These species can provide oxophilic interfacial sites that facilitate water adsorption and dissociation during the Volmer step, thereby promoting alkaline HER kinetics [39, 52, 72, 73, 74].

### 2.4 Initial Surface Stability Assessment

Stability tests performed under both potentiostatic and galvanostatic conditions (see Figure S3) demonstrate that all electrodes maintain stable electrochemical behavior during alkaline HER at both 22 and 60 °C. The chronoamperometric and chronopotentiometric responses exhibit no significant fluctuations, indicating good operational durability under continuous electrolysis.

To further investigate the surface morphology of the electrodes after electrochemical measurements, WLI analysis on the electrodes was performed. The WLI images are shown in Figure S4, and the corresponding surface topography parameters are summarized in Table 1. Based on these results, the reference Ni foil exhibits minor changes after HER, showing a slight variation in structure depth from 0.25 to 0.29 µm and interfacial area ratio from 0.80% to 1.17%. For the electrode processed at the lowest fluence (Φ_cum_ = 48.8 J cm^−2^), the surface parameters after the HER were *S*_d_ = 0.26 ± 0.03 µm and *S*_dr_ = 1.14% ± 0.16%. These relatively low roughness parameters are consistent with its lower electrochemical performance. In contrast, the electrode treated at Φ_cum_ = 608.8 J cm^−2^ retains comparable roughness parameters after HER (*S*_d_ = 0.59 ± 0.09 µm and *S*_dr_ = 11.13% ± 1.23%), while the electrode treated at Φ_cum_ = 2439.3 J cm^−2^ shows a slight increase in structure depth and interfacial area ratio, reaching values of *S*_d_ = 2.51 ± 0.05 µm and *S*_dr_ = 107.33% ± 6.11%, respectively. This behavior suggests that the electrochemical process may further promote surface activation by exposing additional active regions rather than inducing surface degradation.

Taken together, the chronoamperometric and chronopotentiometric responses, combined with the post‐test WLI measurements, indicate that the DLIP‐structured electrodes retained their electrochemical response and characteristic surface topography during the investigated HER tests. The absence of pronounced current or potential fluctuations and the preservation of the structure depth and developed interfacial area suggest no substantial surface degradation under the applied conditions.

## 3 Conclusions

This study demonstrates that DLIP can be used to systematically modify the surface topography of Ni electrodes for the hydrogen evolution reaction in alkaline media. Periodic line‐like structures were produced using cumulative fluences ranging from 48.8 to 2439.3 J cm^−2^. Increasing the cumulative fluence increased the structure depth and developed the interfacial area, reaching 2.35 ± 0.04 μm and 87.29% ± 0.21%, respectively, at the highest fluence.

The electrochemical measurements showed that the DLIP‐induced topographical modifications enhanced the HER performance of the Ni electrodes at both 22 and 60 °C. At 22 °C, the electrode processed at 609.8 J cm^−2^ exhibited the best HER performance, achieving an overpotential reduction of 28% relative to the untreated reference. In contrast, at 60 °C, the 2439.3 J cm^−2^ electrode showed the best performance, with an overpotential reduction of approximately 53% compared with that of the untreated reference.

This improvement was accompanied by a pronounced increase in the electrochemically active surface area, from 2.24 to 31.04 cm^2^ at 22 °C and from 1.95 to 74.84 cm^2^ at 60 °C, as determined at an applied overpotential of 100 mV. The impedance results further indicated more favorable interfacial charge‐transfer behavior for the structured electrodes.

The comparison between 22 and 60 °C demonstrates that the beneficial effect of laser structuring becomes more pronounced at elevated temperature. Chronoamperometric and chronopotentiometric measurements additionally confirmed stable electrochemical behavior under the investigated HER conditions. These results establish a quantitative relationship between DLIP‐generated surface topography, electrochemically accessible area, and HER performance, supporting DLIP as a suitable surface‐engineering strategy for Ni electrodes used in alkaline electrolysis.

## 4 Experimental Section

### 4.1 Material Preparation

Nickel foils (99.95% purity, Goodfellow, USA) were used as electrode substrates. To ensure a contaminant‐free surface prior laser treatment, the foils were cleaned in an ultrasonic bath immersed in ethanol for 10 min at 25 °C and subsequently dried using compressed air. The samples had dimensions of 10 × 10 mm^2^, 125‐μm thickness, and an initial average surface roughness (*S*_a_) of 0.25 μm.

### 4.2 Laser Structuring Experiments

The laser structuring of the nickel surfaces was performed in a DLIP system using a picosecond (ps) pulsed solid‐state laser source (neoMOS, neoLASE GmbH, Hannover, Germany). The DLIP setup consisted of a two‐beam optical head (ELIPSYS, SurFunction GmbH, Germany) that produces periodic line‐like structures with a spatial period (Λ) of 5.4 µm. The schematic setup of the configuration is shown in Figure 6a.

**FIGURE 6:**
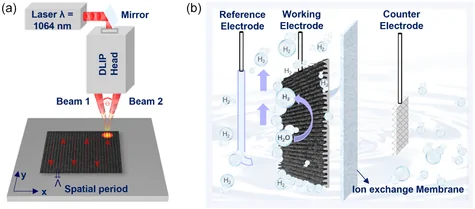
Schematic representation of (a) DLIP laser configuration of two beams producing an interference pattern and a spatial period of Λ = 5.4 µm, and (b) electrochemical setup using three‐electrode configuration cell under alkaline conditions analyzing the laser structured electrode as the working electrode.

The structuring process was conducted at a wavelength (*λ*) of 1064 nm, with a pulse duration (*τ*) of 70 ps and a repetition rate (*ƒ*) of 1 kHz. The laser spot covered an elliptical area of 136 µm × 360 µm (*d*_*x*_ × *d*_*y*_) on the material surface. During processing, the laser texturing parameters were systematically varied by modifying the pulse‐to‐pulse distance, while maintaining the hatch distance (HD = 48.6 μm) and the number of scans (*n* = 1) constant. Using a laser power of 635 mW, a laser fluence corresponded to 1.65 J cm^−2^ per pulse was achieved. Three structures were fabricated with Φ_cum_ of 48.8, 609.8, and 2439.3 J cm^−2^.

### 4.3 Surface Characterization

Surface morphology characterization of the nickel foils was carried out using scanning electron microscopy (SEM) operating at an acceleration voltage of 2 kV (DSM 982 Gemini, Carl Zeiss AG, Jena, Germany). In addition, the surface topography was analyzed by white‐light interferometry (WLI, Sensofar S‐Neox, Spain) using a 50× objective, providing a vertical resolution of 1 nm and a lateral resolution of 300 nm. The surface roughness parameters were evaluated by an advanced surface software (SensoMAP 7.3, Spain).

### 4.4 Electrochemical Analysis

The electrochemical experiments were conducted in a three‐electrode configuration using two cells separated by a perfluorinated cation‐exchange membrane (Nafion 117). In the first compartment, the nickel foil served as the working electrode, while a Hg/HgO/1 M KOH electrode, positioned in a Haber–Luggin capillary approximately 1 mm from the working electrode, was employed as the reference electrode to minimize the uncompensated resistance. A schematic representation of the electrochemical setup is provided in Figure 6b. In the second compartment, a platinum mesh (10 mm × 30 mm) was employed as the counter electrode. The electrodes were connected to a potentiostat (Interface 1010T, Gamry, USA), and the electrochemical data were analyzed using an electrochemical analysis software (Gamry Echem Analyst). The electrolyte used for the experiment was 1 M NaOH solution (99.99% purity, VWR Chemicals), and the pH was ~13.

Prior to the measurement, the electrodes were cleaned in deionized water using an ultrasonic bath for 10 min to remove possible surface contaminants. To define the electrochemical studied area, the material out of the investigated surface were insulated using a chemically resistant polymer (2‐component epoxy adhesive, UHU PLUS). Dissolved oxygen was removed from the electrolyte by bubbling nitrogen gas for 20 min before each experiment [75]. All experiments were conducted inside a Faraday cage at room temperature (22 ± 2 °C) and elevated temperature (60 ± 2 °C).

The HER performance of the electrodes was evaluated following an electrochemical protocol adapted from the literature [68, 76, 77]. As part of this protocol, the electrodes were initially activated by doing cyclic voltammetry in order to clean and stabilize the surface. Subsequently, LSV were carried out in the potential range from 0.0 to −1.3 V (vs. Hg/HgO), while EIS measurements were performed at 100, 200, and 300 mV to evaluate the electrocatalytic activity and charge‐transfer kinetic of the electrodes. The LSV curves and the corresponding Tafel analysis were iR‐corrected using the solution resistance (*R*_s_) extracted from the EIS measurements. The EIS data were analyzed by fitting the experimental results to the corresponding equivalent electrical circuits. For the reference Ni electrode, the impedance results were fitted using a Randles equivalent circuit incorporating a constant phase element, whereas the DLIP‐structured electrodes were analyzed using an equivalent circuit with two‐time constants. From these fittings, the CPE, *R*_s_, *R*_ct_, and *α* parameters were extracted to calculate *C*_dl_. The ECSA at different applied potentials was subsequently estimated by dividing the calculated *C*_dl_ by the specific capacitance of nickel electrodes in alkaline media (*C*_s_ = 40 μF cm^−2^) [77]. Finally, the durability and electrochemical stability of the samples were investigated by chronoamperometry and chronopotentiometry for 40 min.

## Funding

This work was supported by the European Regional Development Fund (100757494).

## Supporting information

Supplementary Material

## Data Availability

The data that support the findings of this study are available from the corresponding author upon reasonable request.
