# Supplementary material for: Laser‐Structured Nickel Electrodes for Enhanced Hydrogen Evolution Kinetics in Alkaline Media at Elevated Temperature
Source: Small Sci. 2026 Oct 25;6(11):e70426. doi: 10.1002/smsc.70426 (PMC13696023; doi:10.1002/smsc.70426)
Supplement: Supplementary file 1 [file SMSC-6-e70426-s001.pdf]

Supporting information

Laser-Structured Nickel Electrodes for Enhanced Hydrogen Evolution Kinetics in Alkaline Media at Elevated Temperature

Mayla Selene Barrientos, Lis Geraldine Zschach, Robert Baumann*, Andrés Fabián Lasagni,

M. Barrientos, L. G. Zschach, R. Baumann, A. F. Lasagni
Institut für Fertigungstechnik, Technische Universität Dresden, George-Bähr-Str. 3c, 01069 Dresden, Germany
A. F. Lasagni,
Fraunhofer-Institut für Werkstoff- und Strahltechnik IWS, Winterbergstr. 28, 01277 Dresden, Germany
*E-mail: robert.baumann1@tu-dresden.de;

Laser Structuring Process

Equations (1-4) used to calculate the laser parameters applied for the fabrication of line-like interference patterns in the DLIP structuring process:

$$\Phi_{\mathrm{cum}} = \frac{\mathrm{E}_p}{A_{spot}} \cdot N_{pulses}, \quad \text{(S1)}$$

$$\mathrm{E}_p = P \cdot \mathrm{f}, \quad \text{(S2)}$$

$$A_{spot} = \pi \cdot \left(\frac{\mathrm{d}_x}{2}\right) \cdot \left(\frac{\mathrm{d}_y}{2}\right), \quad \text{(S3)}$$

$$N_{pulses} = \mathrm{d}_x \cdot \frac{f}{v} \cdot \frac{\mathrm{d}_y}{HD}, \quad \text{(S4)}$$

where, Φ_{cum} is the total applied laser energy density, called cumulated laser fluence, E_p is the pulse energy, N_{pulses} is the total number of pulses used to treat a specific area, P the laser power, f the repletion rate of the laser source (frequency), d_x and d_y are the spot diameter in x and y axis, v the translation speed of the stage holding the Ni foil and HD is the hatch distance.

Table S1. Laser parameters used for the DLIP structuring process the electrodes.

**Process speed *v*** [mm s^{-1}]	**Pulse to pulse distance Δ_x** [µm]	**Pulse number N_{pulses}** [-]	**Cum. fluence Φ_{cum}** [J cm^{-2}]
34.1	30.0	29.52	48.8
2.7	3.0	369.03	609.8
0.7	0.7	1476.13	2439.3

Table S2. Equivalent electrical circuit (EEC) parameters extracted from EIS at 100 mV for unstructured reference electrode and DLIP structured electrodes with cumulated fluences of 48.8 J cm^{-2}, 609.8 J cm^{-2}, 2439.3 J cm^{-2} in 1 M NaOH solution at both temperatures (22- 60 °C).

**Temperature**	**22 °C**				**60 °C**			
**Electrical Circuit Elements**	**Reference (Ni foil)**	**Φ_{cum} = 48.8 J cm^{-2}**	**Φ_{cum} = 609.8 J cm^{-2}**	**Φ_{cum} = 2439.3 J cm^{-2}**	**Reference (Ni foil)**	**Φ_{cum} = 48.8 J cm^{-2}**	**Φ_{cum} = 609.8 J cm^{-2}**	**Φ_{cum} = 2439.3 J cm^{-2}**
**R_s** [Ω cm²]	2.920	2.322	2.091	0.565	1.399	0.921	0.337	0.850
**R_O** [Ω cm²]	-	420.6	92.6	336.0	-	112.6	163.1	106.8
**CPE_O** [S s^α]	-	1.23×10^{-4}	5.38×10^{-4}	1.57×10^{-3}	-	1.80×10^{-4}	6.54×10^{-4}	3.28×10^{-3}
**α_O** [-]	-	1.000	1.000	0.708	-	0.848	0.7932	0.6502
**R_{ct}** [Ω cm²]	106000	10800	10500	4260	25540	4730	2954	8614
**CPE_{dl}** [S s^α]	7.81×10^{-5}	4.19×10^{-4}	6.12×10^{-4}	1.48×10^{-3}	7.34×10^{-5}	4.22×10^{-4}	6.27×10^{-4}	5.59×10^{-4}
**α_{cl}** [-]	0.885	0.718	0.762	1.000	0.845	0.787	0.912	0.922

Table S3. Equivalent electrical circuit (EEC) parameters extracted from EIS at 200 mV for unstructured reference electrode and DLIP structured electrodes with cumulated fluences of 48.8 J cm^{-2}, 609.8 J cm^{-2}, 2439.3 J cm^{-2} in 1 M NaOH solution at both temperatures (22- 60 °C).

**Temperature**	**22 °C**				**60 °C**			
**Electrical Circuit Elements**	**Reference (Ni foil)**	**Φ_{cum} = 48.8 J cm^{-2}**	**Φ_{cum} = 609.8 J cm^{-2}**	**Φ_{cum} = 2439.3 J cm^{-2}**	**Reference (Ni foil)**	**Φ_{cum} = 48.8 J cm^{-2}**	**Φ_{cum} = 609.8 J cm^{-2}**	**Φ_{cum} = 2439.3 J cm^{-2}**
**R_s** [Ω cm²]	1.66	2.192	2.049	0.6	1.445	0.894	0.315	1.015
**R_O** [Ω cm²]	-	453.5	472.1	370	-	84.15	109	313.3
**CPE_O** [S s^α]	-	8.06×10^{-5}	3.75×10^{-4}	1.37×10^{-3}	-	1.65×10^{-4}	6.94×10^{-4}	1.94×10^{-3}
**α_O** [-]	-	1	0.778	0.715	-	0.847	0.7852	0.7265
**R_{ct}** [Ω cm²]	162000	18900	8240	8120	36030	6620	9649	4375
**CPE_{dl}** [S s^α]	7.19×10^{-5}	3.26×10^{-4}	4.88×10^{-4}	8.59×10^{-4}	4.41×10^{-5}	3.00×10^{-4}	3.80×10^{-4}	8.87×10^{-4}
**α_{cl}** [-]	0.898	0.713	0.959	0.988	0.883	0.789	0.861	1.000

Table S4. Equivalent electrical circuit (EEC) parameters extracted from EIS at 300 mV for unstructured reference electrode and DLIP structured electrodes with cumulated fluences of 48.8 J cm^{-2}, 609.8 J cm^{-2}, 2439.3 J cm^{-2} in 1 M NaOH solution at both temperatures (22- 60 °C).

Temperature	22 °C				60 °C			
Electrical Circuit Elements	Reference (Ni foil)	Φ_{cum} = 48.8 J cm^{-2}	Φ_{cum} = 609.8 J cm^{-2}	Φ_{cum} = 2439.3 J cm^{-2}	Reference (Ni foil)	Φ_{cum} = 48.8 J cm^{-2}	Φ_{cum} = 609.8 J cm^{-2}	Φ_{cum} = 2439.3 J cm^{-2}
R_s [Ω cm²]	2.604	2.370	1.951	0.577	1.428	0.877	0.2453	0.8018
R_O [Ω cm²]	-	799	374	242	-	87	330	245
CPE_O [S s^{α}]	-	7.36×10^{-5}	2.58×10^{-4}	9.78×10^{-4}	-	1.35×10^{-4}	3.99×10^{-4}	1.98×10^{-3}
α_O [-]	-	0.858	0.788	0.730	-	0.840	0.763	0.681
R_{ct} [Ω cm²]	227000	16800	11800	13900	46940	8280	12900	6503
CPE_{dl} [S s^{α}]	6.72×10^{-05}	2.79×10^{-4}	3.52×10^{-4}	6.00×10^{-4}	4.90×10^{-5}	2.31×10^{-4}	2.89×10^{-4}	5.77×10^{-4}
α_{cl} [-]	0.879	0.811	0.905	0.928	0.876	0.801	0.973	0.984

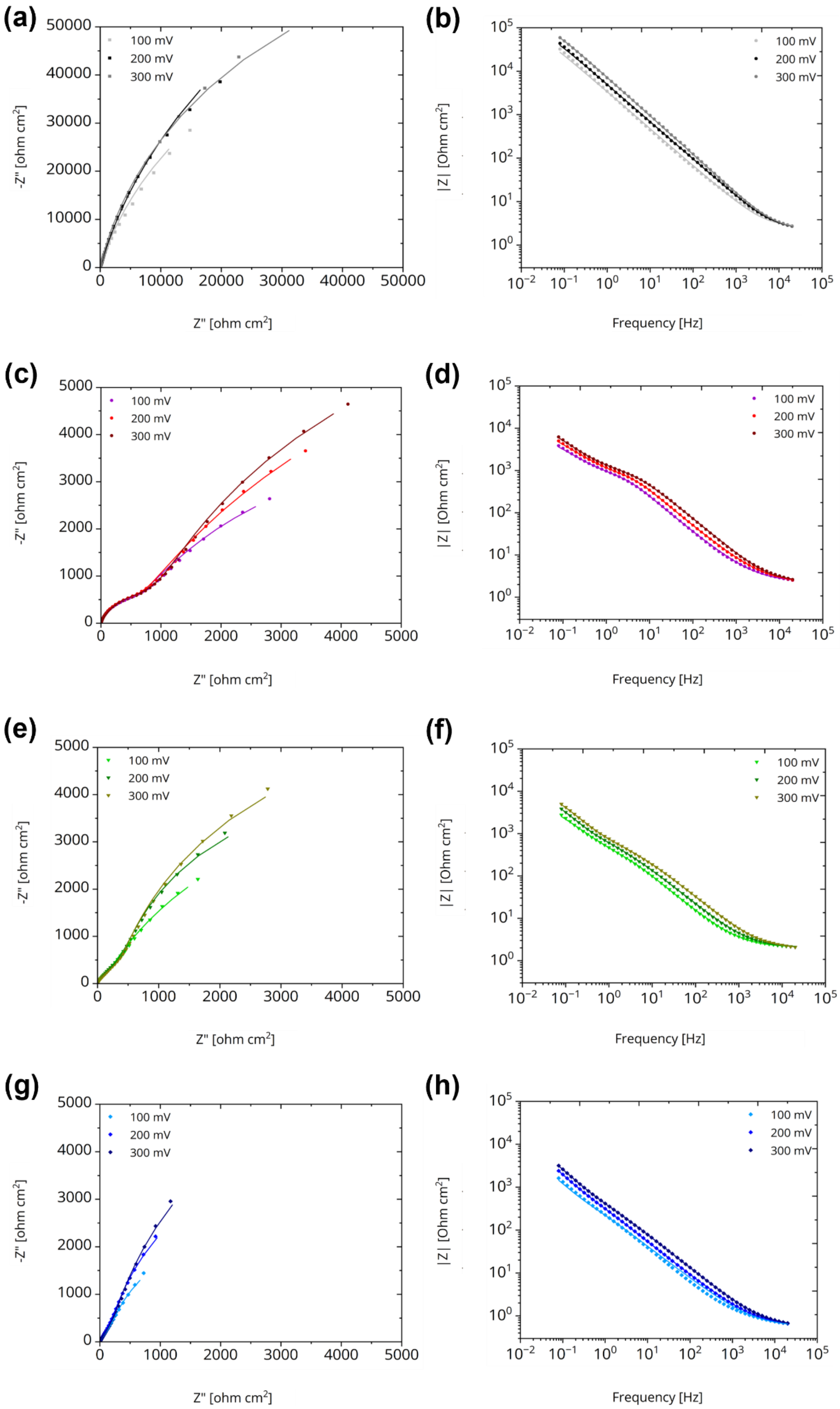

Figure S1. Electrochemical impedance spectroscopy (EIS) at different applied potentials in 1 M NaOH solution at 22°C for unstructured reference electrode (a-b) and DLIP structured electrodes with cumulated fluences of 48.8 J cm^{-2} (c-d), 609.8 J cm^{-2} (e-f), 2439.3 J cm^{-2} (g-h).

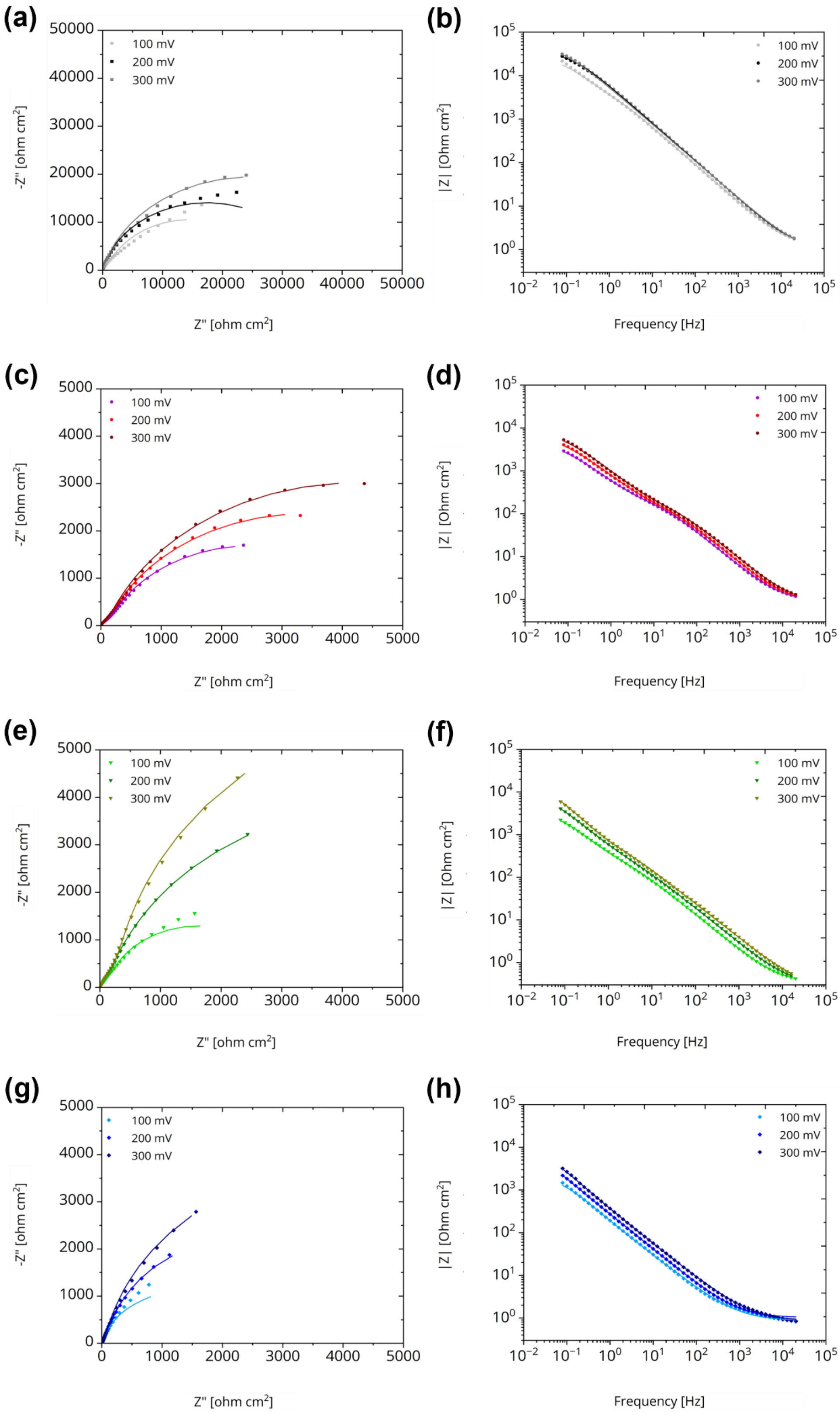

Figure S2. Electrochemical impedance spectroscopy (EIS) at different applied potentials in 1 M NaOH solution at 60°C for unstructured reference electrode (a-b) and DLIP structured electrodes with cumulated fluences of 48.8 J cm^{-2} (c-d), 609.8 J cm^{-2} (e-f), 2439.3 J cm^{-2} (g-h).

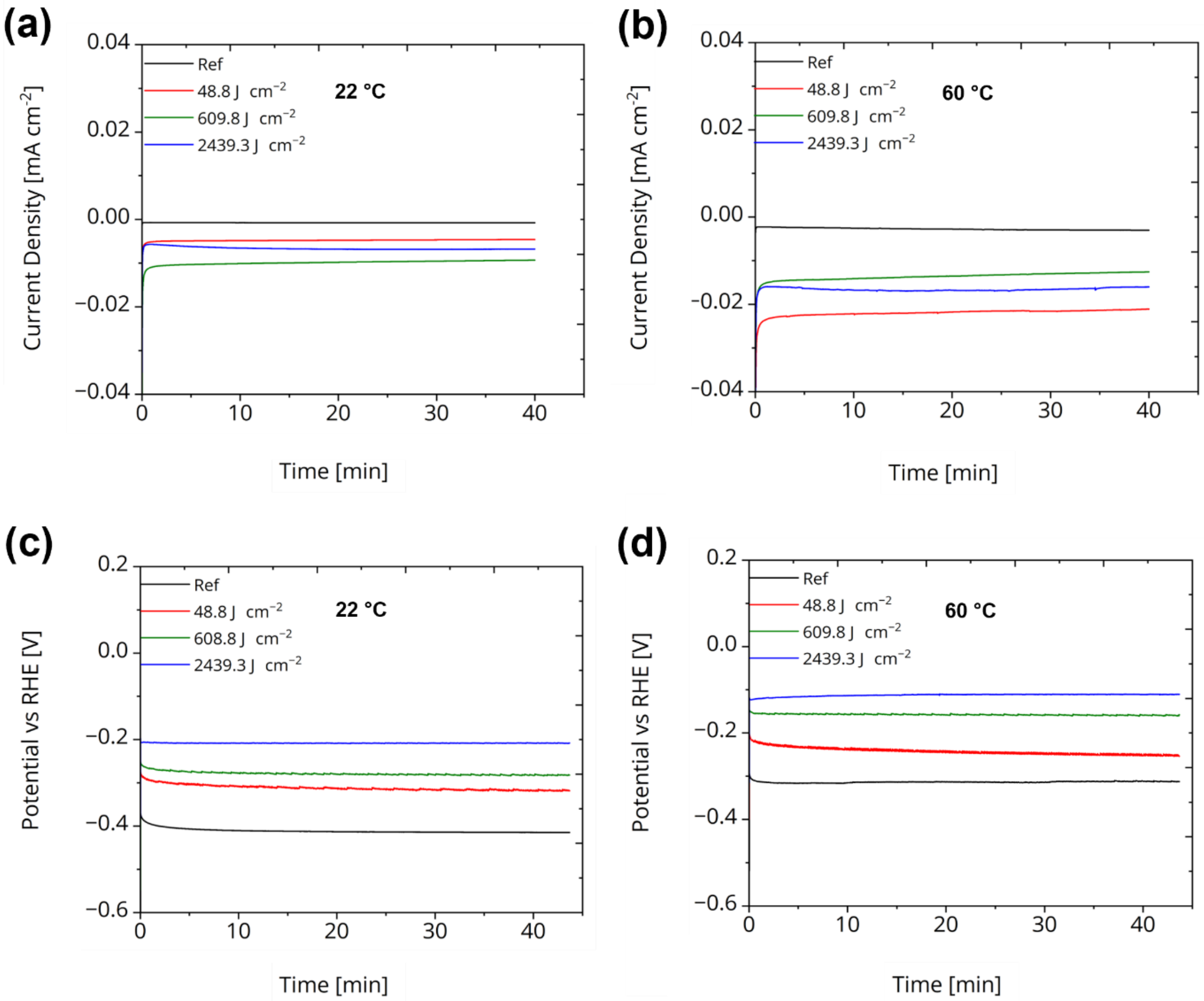

Figure S3. Chronoamperometric plots (a-b) and chronopotentiometric plots (a-b) in 1 M NaOH solution at both temperatures (22- 60 °C) for unstructured reference electrode and DLIP structured electrodes with cumulated fluences of 48.8 J cm^{-2}, 609.8 J cm^{-2}, 2439.3 J cm^{-2}.

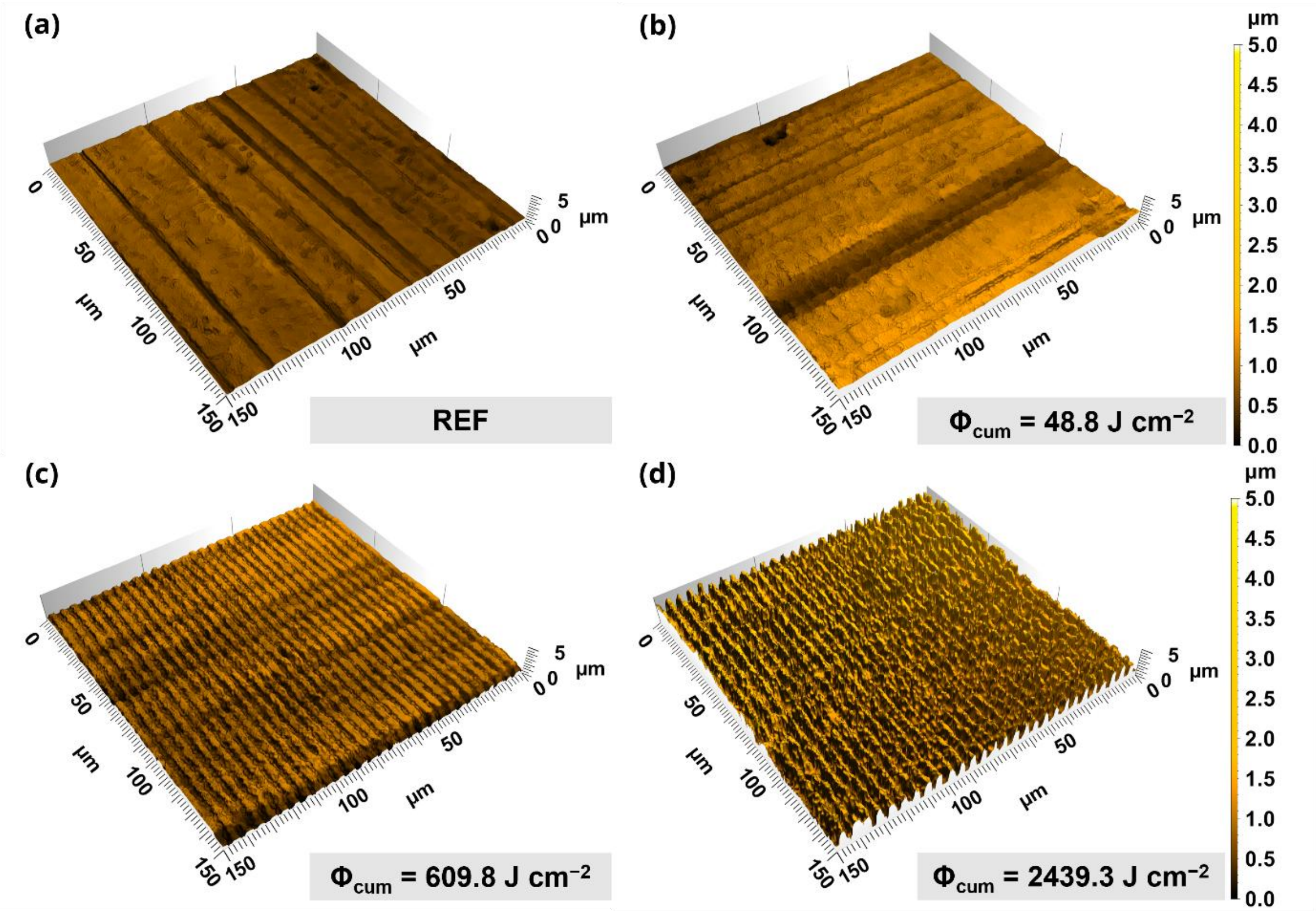

Figure S4 WLI images after HER of (a) unstructured reference Ni foil and line-like DLIP periodic Ni structured with cumulated fluence of (b) 48.8 J cm^{-2} (c) 609.8 J cm^{-2} and (d) 2439.3 J cm^{-2}. The resulting periodic structures reveal a spatial period (Λ_{DLIP}) of 5.4 µm.
